# Outbreak of human monkeypox, Democratic Republic of Congo, 1996 to 1997.

**DOI:** 10.3201/eid0703.010311

**Published:** 2001

**Authors:** Y J Hutin, R J Williams, P Malfait, R Pebody, V N Loparev, S L Ropp, M Rodriguez, J C Knight, F K Tshioko, A S Khan, M V Szczeniowski, J J Esposito

**Affiliations:** Centers for Disease Control and Prevention, Atlanta, Georgia 30333, USA.

## Abstract

Human monkeypox is a zoonotic smallpox-like disease caused by an orthopoxvirus of interhuman transmissibility too low to sustain spread in susceptible populations. In February 1997, 88 cases of febrile pustular rash were identified for the previous 12 months in 12 villages of the Katako-Kombe Health Zone, Democratic Republic of Congo (attack rate = 22 per 1,000; case-fatality rate = 3.7%). Seven were active cases confirmed by virus isolation. Orthopoxvirus- neutralizing antibodies were detected in 54% of 72 patients who provided serum and 25% of 59 wild-caught animals, mainly squirrels. Hemagglutination-inhibition assays and Western blotting detected antibodies in 68% and 73% of patients, respectively. Vaccinia vaccination, which protects against monkeypox, ceased by 1983 after global smallpox eradication, leading to an increase in the proportion of susceptible people.


### Outbreak of Human Monkeypox, Democratic Republic of Congo, 1996–1997

**Yvan J.F. Hutin,* R. Joel Williams,* Philippe Malfait,† Richard Pebody,† Vladamir N. Loparev,* Susan L. Ropp,* Mariangelli Rodriguez,* Janice C Knight,* Florimont K. Tshioko,‡ Ali S Khan,* Mark V. Szczeniowski,‡ and Joseph J. Esposito***

*Centers for Disease Control and Prevention, Atlanta, Georgia, USA;
†European Programme for Intervention Epidemiology Training, Brussels, Belgium;
and ‡World Health Organization, Geneva, Switzerland

Human monkeypox is a zoonotic smallpox-like disease caused by an orthopoxvirus of interhuman transmissibility too low to sustain spread in susceptible populations. In February 1997, 88 cases of febrile pustular rash were identified for the previous 12 months in 12 villages of the Katako-Kombe Health Zone, Democratic Republic of Congo (attack rate = 22 per 1,000; case-fatality rate = 3.7%). Seven were active cases confirmed by virus isolation. Orthopoxvirus-neutralizing antibodies were detected in 54% of 72 patients who provided serum and 25% of 59 wild-caught animals, mainly squirrels. Hemagglutination-inhibition assays and Western blotting detected antibodies in 68% and 73% of patients, respectively. Vaccinia vaccination, which protects against monkeypox, ceased by 1983 after global smallpox eradication, leading to an increase in the proportion of susceptible people.

Human monkeypox, a sporadic smallpox-like zoonotic viral exanthema that occurs in the rain forests of Central and West Africa, was discovered in 1970 (1-3). The illness is caused by an orthopoxvirus, monkeypox virus, which was first isolated from primate tissues (4). Animal antibody surveys in the Democratic Republic of Congo (DRC; former Zaire) suggested that squirrels play a major role as a reservoir of the virus and that humans are sporadically infected (3,5,6). Human-to-human transmission occurs with an incubation period of 12 days (range 7-21 days) (3).

After smallpox eradication, surveillance for human monkeypox from 1981 to 1986 in the DRC identified 338 cases (67% confirmed by virus culture). The case-fatality rate was 9.8% for persons not vaccinated with vaccinia (smallpox) vaccine, which was about 85% efficacious in preventing human monkeypox (3,7). The secondary attack rate in unvaccinated household members was 9.3%, and 28% of case-patients reported an exposure to another case-patient during the incubation period. Transmission chains beyond secondary were rare (8,9). A mathematical model to assess the potential for monkeypox to spread in susceptible populations after cessation of vaccinia vaccination indicated that person-to-person transmission would not sustain monkeypox in humans without repeated reintroduction of the virus from the wild (7).

In 1996, 71 suspected human monkeypox cases were reported from the Katako-Kombe Health Zone, Kasai Oriental, DRC. These initial reports suggested predominant person-to-person transmission and prolonged chains of transmission. Two cases were confirmed by monkeypox virus isolation from lesion material (10). In February 1997, an investigation was initiated (11). Our report describes epidemiologic observations and laboratory results supporting the conclusion that repeated animal reintroduction of monkeypox virus is needed to sustain the disease in the local human population.

Address for correspondence: Joseph J. Esposito, Director, WHO Collaborating Center for Smallpox and Other Poxvirus Infections, Centers for Disease Control and Prevention, 1600 Clifton Road, MS G18, Atlanta, GA 30333, USA; fax: 404-639-3111; e-mail: jesposito@cdc.gov

### Methods

### Epidemiologic and Clinical Studies

Before civil unrest in the area curtailed the study, a dwelling-to-dwelling search was conducted for cases that occurred from February 1996 and February 1997 in 12 villages (total population 4,057) in the Katako-Kombe Health Zone, located around Akungula, a village reported to be the epicenter of the current outbreak. A clinical case of monkeypox was defined as the occurrence of fever with a rash recognized as being similar to that in a reference photo provided by the World Health Organization. Monkeypox cases were classified as active until desquamation of the rash. After desquamation, cases were identified retrospectively by interview and examination for residual scars. Onset dates were estimated by using local event calendars.

Patients (or their adult respondent) who agreed to participate were queried by using a standardized data collection instrument to obtain information on demographics, signs and symptoms of disease, exposures to wild animals, presence of a smallpox vaccination scar, and exposure to another patient. Consenting participants underwent a physical examination, and a blood sample for serum was obtained.

### Animal Studies

Local trappers were asked to capture and bring to the study veterinarian wild animals, especially rodents, squirrels, and nonhuman primates, for which the trappers were paid as an incentive. Animals were processed in a field laboratory (12), identified to genus and species, and bled for serum. Representative voucher specimens of each animal were preserved in 10% formalin for definitive identification.

### Laboratory Studies

Human and animal sera were clarified by low-speed centrifugation, immediately stored in liquid nitrogen, and shipped to the Centers for Disease Control and Prevention (CDC) in Atlanta. At CDC, an aliquot of each serum was heated at 56ºC for 30 minutes, the following tests were performed: 1) vaccinia virus hemagglutination-inhibition (HAI) assay (13); 2) monkeypox 50% plaque-reduction neutralization assay (13); and 3) Western blot assays for immunoglobulin G (IgG) against monkeypox antigens essentially by using Towbin's and colleagues' methods adapted to mini-transblot and multiscreen apparatuses (Bio-Rad, Hercules, CA) (14). Western blotting used selected human sera and antigen preparations that consisted of a soluble antigen (20x culture medium concentrate) from monkeypox virus-infected Vero cells (15). Positive controls consisted of sera collected in the 1980s from convalescent-phase monkeypox cases from the prospective study in the DRC and from vaccinia-vaccinated persons; negative controls consisted of sera collected in the 1980s from DRC inhabitants with no history of vaccinia vaccination or monkeypox. Human sera were also tested for antibodies against varicella virus by using kits to detect human IgM or IgG by enzyme-linked immunosorbent assays (ELISA; Wampole Laboratories, Cranberry, NJ). Samples of crusted scab or pustule lesion were cultured for monkeypox virus using the monkey cell lines Vero, LLCMK-2, or OMK (13,16), and assayed by polymerase chain reaction (PCR) amplification for monkeypox virus-specific DNA (16,17) and for varicella virus gene 1 (P. Pellett, pers. comm.). In addition, the gene encoding the hemagglutinin (HA) protein of selected monkeypox isolates was sequenced by fluorescence-based methods (Applied Biosystems, Inc., Foster City, CA). Available duplicate coded sera were tested anonymously in Kinshasa, DRC, for antibodies against HIV (Vironostika Human Form II ELISA, Organon Teknika, Denmark).

The relatedness of isolates was examined by comparing DNA restriction endonuclease patterns with patterns of previously mapped monkeypox virus isolates (18) and by comparing the hemagglutinin gene sequences with cognate sequences of other monkeypox isolates.

### Statistical Methods

Attack rates were calculated by using a census conducted during the dwelling-to-dwelling case search; information on the age and sex of each person living in Akungula was also obtained. Secondary attack rates within households were calculated by dividing the number of cases that occurred 7 to 21 days following one or more index cases in a household (first-generation secondary cases) by the total number of household members, excluding index cases. Confidence intervals (CI) for proportions were calculated with exact methods and compared with Fisher's exact tests as appropriate by using Epi-Info software (19).

### Results

### Epidemiologic and Clinical Studies

Eighty-eight clinical cases (7 active and 81 retrospectively identified) were discovered in 9 of the 12 villages (attack rate 22 per 1,000). Fifty (56.8%) of 88 case-patients were male; the median age at onset was 10 years (quartiles 5-19; range 1 month to 62 years).

The number of clinical cases reported per month increased from February to August 1996, followed by a decline until January 1997 and a resurgence of cases in February 1997 (Figure 1). Attack rates in the villages ranged from zero to 105 per 1,000 population in Akungula, where 42 cases were identified in 14 of the 62 village households.

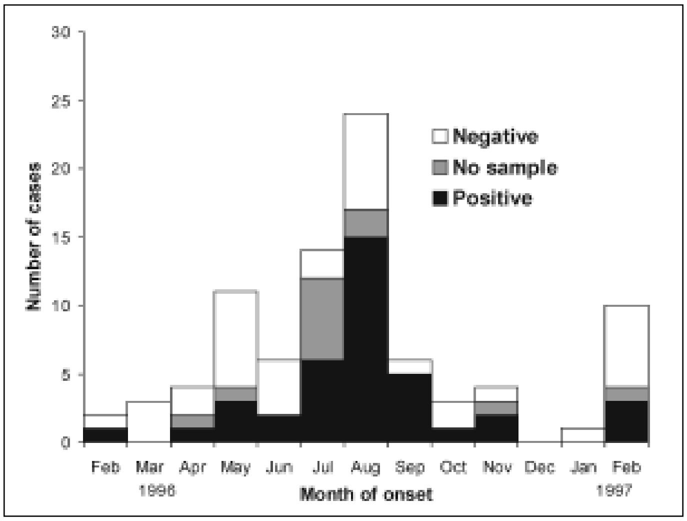

Figure 1. Human monkeypox cases by month of onset in 12 villages, according to results of neutralization assay, Katako-Kombe Health Zone, February 1996 to February 1997.

In Akungula, where the age and sex distributions of the population were available, the attack rate was identical for males and females, but was higher (30 of 206 = 146/1,000) in children <15 years of age than in persons 15 to 24 years old (4 of 85 = 47 /1,000) and ≥25 years of age (8 of 109 = 73/1,000).

Of 78 retrospectively identified case-patients for whom information was available, 40 (51.3%) reported swelling consistent with lymphadenopathy. All 75 examined patients had scars from a rash (median 50; range 4 to 830; standard deviation 179). Thirteen (15.5%) of 84 patients for whom information was available had a vaccination scar on the upper left arm compatible with prior vaccinia vaccination; all patients were ≥20 years of age. Alopecia was seen in three cases with acute rash illness. Three deaths occurred among 81 of the 88 cases for which follow-up information was available (3.7% case-fatality rate), all in children <3 years of age who died within 3 weeks of rash onset. No information was available to attribute the deaths to monkeypox, superinfection, or other cause.

Seven case-patients (six in a single household) had active disease at the time of the investigation. Each had lymphadenopathy and more than 100 crusty skin lesions. Five had a rash on the soles and palms. Sixty-two (73%) of 85 case-patients for whom information was available reported exposure to another patient 7 to 21 days before onset of their illness. The remaining 23 (27%) patients reported either no exposure to other cases (n=13; 15%) or an exposure to another case from 0 to 6 days before onset of illness (n=10; 12%). Dates of onset for patients who reported no exposure to other cases during the incubation period were distributed throughout the study period (11). Exposures during the incubation period that were reported by all six patients who resided in a single household and who had active disease at the time of this investigation included exposure to a patient within the household and eating monkey, gazelle, pig, and rat.

The 88 clinical cases were identified for 39 households, in which 297 persons resided (overall attack rate in affected households = 30%). For 240 household members for whom information was available, rash that met the clinical case definition developed in 20 in 7 to 21 days after exposure to ≥1 index cases in a household (household secondary attack rate 8.3 per 100; 95% CI 5.2% to 12.6%).

Eating wild animals was common for all patients. However, patients who had no exposure to other case-patients during the incubation period were more likely to eat porcupine at least once a month (Table 1). All queried participants reported trapping animals less frequently.

### Laboratory Studies

Human monkeypox was confirmed in all seven active cases by virus isolation and monkeypox virus-specific DNA amplification from skin lesion samples; antibodies against orthopoxvirus were detected in two by neutralization assay, in three by HAI assay, and in six by Western blotting. In addition, IgM antibodies against varicella virus were detected in five active cases in patients who lived in the same household. Varicella virus DNA was detected in lesion material from two of these patients.

Table 1. Exposure to wild animals reported by monkeypox case-patients according to history of exposure to another case-patient, Katako-Kombe Health zone, February 1996 to February 1997

| Exposures reported to occur usually on a monthly basis | Cases without exposure to other case-patients during incubation (N=23) (#) | (%) | Cases with exposure to other case-patients during incubation (N=62) (#) | (%) | p value |
|---|---|---|---|---|---|
| Exposure to squirrels | | | | | |
| Trapping | 2 | 8.7 | 9 | 14.8 | 0.79 |
| Exposure to raw meat | 11 | 47.8 | 33 | 54.1 | 0.78 |
| Eating | 19 | 86.4 | 52 | 85.2 | 1.00 |
| Exposure to monkeys | | | | | |
| Trapping | 1 | 4.3 | 4 | 6.6 | 1.00 |
| Exposure to raw meat | 16 | 69.6 | 34 | 55.7 | 0.36 |
| Eating | 21 | 91.3 | 58 | 95.1 | 0.61 |
| Exposure to rats | | | | | |
| Trapping | 5 | 21.7 | 18 | 29.5 | 0.66 |
| Exposure to raw meat | 13 | 56.5 | 40 | 65.6 | 0.60 |
| Eating | 22 | 95.7 | 55 | 90.2 | 0.66 |
| Exposure to porcupines | | | | | |
| Trapping | 1 | 4.3 | 3 | 4.9 | 1.00 |
| Exposure to raw meat | 12 | 52.2 | 24 | 39.3 | 0.41 |
| Eating | 22 | 95.7 | 42 | 68.9 | 0.02 |
| Exposure to gazelles | | | | | |
| Trapping | 1 | 4.3 | 2 | 3.3 | 1.00 |
| Exposure to raw meat | 14 | 56.5 | 28 | 45.9 | 0.53 |
| Eating | 21 | 91.3 | 47 | 77.0 | 0.21 |

Seventy-two of the 81 retrospectively identified cases provided a serum sample, although 2 were low in volume. Orthopoxvirus antibodies were detected by neutralization assay in 39 (54%, Figure 1) of the 72 sera; by HAI in 51 (73%) of 70 sera, and by a new, nonvalidated Western blot test in 49 (68%) of 72 sera. Thirty-eight (54%) of 70 available sera were positive by all three tests, 45 (64%) were positive by two tests, and 62 (89%) were positive by at least one test. Western blotting and HAI test results were concordant for 79% of the sera; Western blotting and neutralization test results agreed for 83% of the sera; and HAI and neutralization test results agreed for 79% of the sera. Fifty-five (76%) of the 72 patients who provided serum had detectable varicella IgG antibodies, 1 had varicella IgM, and none showed detectable HIV antibodies.

Based on DNA assays (16-18), monkeypox virus isolates from the seven active cases appeared virtually identical to each other and very closely related to two isolates from humans in 1996 and an isolate from a squirrel trapped in the Equateur Province of the DRC in 1985. Analysis of sequences coding for the viral HA protein (Figure 2) indicated that viruses isolated in the DRC since 1970 comprised a clade distinguishable from a clade comprising isolates from other African countries and from outbreaks in primate-holding facilities in Europe and the United States. The group of nine isolates from the 1996-97 outbreak, which showed identical HA sequences, and from the squirrel from 1985 constituted a subset of the clade of examined viruses previously isolated in the DRC.

### Animal Studies

Fifty-nine captured animals representing 14 species were tested. Sera from 42% of the various squirrel species, from 16% of Gambian rats, and from an elephant shrew and a domestic pig showed orthopoxvirus neutralizing antibodies (Table 2).

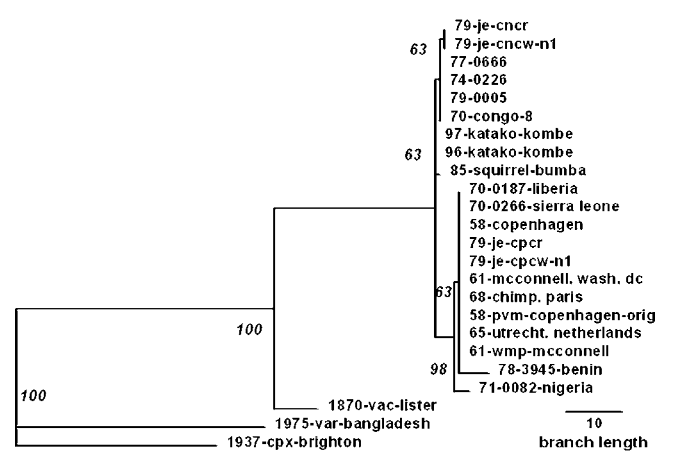

Figure 2. Phylogenetic inference relationships of the open reading frames encoding the viral hemagglutinin protein of various monkeypox virus isolates and selected strains of vaccinia, variola, and cowpox viruses. Nucleotide sequences of polymerase chain reaction-generated amplicons were analyzed using PAUP parsimony analysis software version 3.1.1, as described (10). Parsimony analysis used 5,000 bootstraps and weighted the sequences for a transition-transversion ratio of 2 (bootstrap confidence intervals shown on branches).

Table 2. Species of animals caught in the wild and monkeypox virus plaque reduction neutralization antibody assay results, Katako-Kombe Health Zone, February 23-27, 1997

| Animal species | Common name | No. Tested | Pos. | Proportion pos. |
|---|---|---|---|---|
| *Cercopithecus ascanius* | Spot-nosed monkey | 2 | 0 | 0.0% |
| *Cercopithecus neglectus* | Debrazza monkey | 1 | 0 | 0.0% |
| *Perodicticus potto* | Bosman's potto | 1 | 0 | 0.0% |
| *Galogoides demidovi* | Demidoff galago | 2 | 0 | 0.0% |
| *Genetta rubiginosa* | Genet | 1 | 0 | 0.0% |
| *Sus scrofa* | Domestic pig | 1 | 1 | 100.0% |
| *Praomys jacksoni* | Forest rat | 1 | 0 | 0.0% |
| *Cricetomys emini* | Gambian rat | 19 | 3 | 15.8% |
| *Cavia sp.* | Guinea pig | 3 | 0 | 0.0% |
| *Petrodromus tetradactylus* | Elephant shrew | 3 | 1 | 33.3% |
| *Dendrohyrax arboreus* | Tree hyrax | 1 | 0 | 0.0% |
| *Funisciurus anerythrus* | Thomas's tree squirrel | 4 | 2 | 50.0% |
| *Funisciurus congicus* | Kuhl's tree squirrel | 18 | 7 | 38.9% |
| *Heliosciurus rufobrachium* | Sun squirrel | 2 | 1 | 50.0% |
| Total | | 59 | 15 | 25.4% |

### Conclusion

The epidemiologic and laboratory evidence presented here suggests that this monkeypox outbreak is the largest reported. Epidemiologic features of monkeypox that differed from those described during an intensified prospective study in the DRC in the 1980s included an increase in the proportion of patients reporting an exposure to another case during the incubation period (28% in the 1980s, 73% this study) and the clustering of successive cases within households, with as many as eight consecutive cases in one instance (3,10).

Prospective surveillance in the 1980s in the DRC provided opportunities to observe most patients with active-stage monkeypox; thus, skin lesion samples were available for virus isolation to confirm clinical diagnosis. In contrast, the present study identified most patients retrospectively and relied on serologic testing for confirmation of the diagnosis; only seven clinically active cases could be confirmed by virus isolation and PCR (17) for monkeypox virus. Because monkeypox is the only known indigenous orthopoxvirus of Africa that infects humans systemically, seropositive cases showing genus-specific antibodies can be reasonably interpreted as cases that had a monkeypox virus infection (20). Of 72 retrospectively identified cases tested, 54% had antibodies by plaque-reduction assay, a preferred test for verifying orthopoxvirus infections during smallpox eradication (20,21). However, this neutralization test was only 83% sensitive for the detection of vaccinia vaccine-induced antibodies and may have been negative (3) in some patients with monkeypox virus infection during this study interval. A higher proportion of sera from retrospectively identified cases (73%) tested positive by the HAI test, which had a higher sensitivity (96%) than the neutralization test for the identification of vaccinia-induced antibodies (22). However, the HAI test may be less specific, and antibody titers detected by it decrease rapidly after infection and would be unlikely to be residual from prior vaccination in the small percentage of patients who had been vaccinated (3,21,23). Finally, 72% of sera from retrospectively identified cases tested positive in a new, nonvalidated Western blot orthopoxvirus antibody test for which sensitivity and specificity have not been determined. Chickenpox, which can be mistaken for monkeypox (24), was reported in the area during the study period. Some patients may have had dual infection by monkeypox and varicella viruses. Within one household, five patients from whom monkeypox virus was isolated showed serologic evidence of recent varicella virus infection, and two of those had detectable varicella virus DNA in skin lesion samples. Thus, a substantial percentage of cases may have been chickenpox, thereby explaining why the case-fatality rate from this study (3.7%) was lower than the 9.6% previously reported (3,24). However, the presence of antibodies against orthopoxviruses by three different assays in 54% of patients suggests that varicella virus was not responsible for most cases during the study period.

The 1996-97 crude secondary attack rate of about 8% in households cannot be compared with the 3.7% risk of attack for household contacts from the 1981-1986 study (3) because the proportion of susceptible (unvaccinated) persons in household members increased markedly from 1981-1986 to 1996-97. However, the 1996-97 secondary attack rate within households was similar to the 1981-1986 rates (3) of 9.3% for unvaccinated household members. This similarity is consistent with the high degree of similarity noted in comparing current monkeypox virus isolate DNAs with DNAs of isolates from the DRC in the 1970s, including the HA sequences (Figure 2). While the virus and its person-to-person transmission potential have not changed substantially, the proportion of susceptible persons among exposed household members increased most likely because vaccinia vaccination ceased after smallpox eradication. The reduced herd immunity probably also caused a higher proportion of cases to be attributable to person-to-person transmission, as the increase in the proportion of patients who reported exposure to another patient during the incubation interval would suggest. HIV was not a cofactor of this outbreak because no HIV antibodies were detected in patients' sera.

Exposure to an animal reservoir might have been the source of infection for at least 27% of patients who reported no exposure to other patients during the incubation period. Past animal studies pointed to several species of squirrels as animal reservoirs (5,6). Although the possibility of infection of animals with another unknown indigenous orthopoxvirus cannot be ruled out in animals with neutralizing antibodies, the results of our investigation suggest that, in addition to squirrels, Gambian rats may play a role in monkeypox virus circulation. Villagers ate a number of different animals, and it was impossible to draw any conclusions as to whether any one species was a greater risk factor than any other. However, antibody surveys could be used to evaluate porcupines as a possible reservoir (Table 1), which was not done during our investigation because no porcupines were captured by trappers.

Our study had several limitations, some of which related to the brevity imposed by the civil unrest. Most cases were identified retrospectively and without a case-control group; thus, they could not be confirmed by a serologic test for which the sensitivity and the specificity had been measured using a standard technique and case-control sera. Because information was not available regarding the vaccination status of other members of the patients' households, secondary attack rates within households could not be calculated according to the vaccination status. We did not assess the environmental changes that could have facilitated this outbreak, including increases in household sizes, rates of monkeypox virus infection in the animal population, and a change in rate and type of exposure to wild animals among residents. We were unable to attribute infections in patients who reported an exposure to another patient during the incubation period to person-to-person transmission because some of these patients may also have been exposed to infected animals. Finally, patients with a history of exposure to a patient during the incubation period were the only controls available to generate hypotheses regarding the type of animal exposure possibly associated with infection in patients not reporting an exposure to other patients during the incubation period. This aspect may have caused bias because cases clustered within households where exposures to animals may have been identical and because cases may have been misclassified as to their purported source of infection.

There is no evidence to date that person-to-person transmission alone can sustain monkeypox in the local population. However, our study suggests that in a population with low herd immunity, person-to-person transmission and repeated introduction of virus from the animal reservoir may lead to more and larger clusters of human monkeypox cases in African rain forest areas (25). Further studies are needed to measure the sensitivity and the specificity of the serologic tests currently available so they can be used in the future to better confirm retrospectively identified cases, identify the type of animal or patient exposures associated with acquisition of illness, and evaluate the respective roles of person-to-person and animal-to-human transmission. However, access to the Kasai region has been limited since our study, and little information has become available to better document the current dynamics of monkeypox occurrences or improve World Health Organization recommendations to prevent human monkeypox more substantially.

### Acknowledgments

The authors thank T.P. Muamba, G. Mwema, D. Messinger, K. Kilundu, V.B. Mukinda, L.V. Okito, and V.L. Mangindula for assistance in data collection; L. Otshudi for assistance in setup of the investigation; J.J. Muyembe-Tamfum for HIV testing; A. Moudi; J.A. Stewart and J. Patton for varicella virus testing; A. Moren, W.C. Reeves, C.J. Peters, and D.A. Henderson for critically reading the manuscript; and J. O'Connor for editorial assistance.

The investigation was partly funded by the U.S. Agency for International Development.

Dr. Yvan Hutin was an Epidemic Intelligence Service officer with the Centers for Disease Control and Prevention at the time of this investigation. He is now at the World Health Organization.

### References

1. Marennikova SS, Seluhina EM, Mal'ceva NN, Cimiskjan KL, Macevic GR. Isolation and properties of the causal agent of a new variola-like disease (monkeypox) in man. Bull World Health Organ 1972;46:599-611.
2. Arita I, Jezek Z, Khodakevich L, Ruti K. Human monkeypox: a newly emerged orthopoxvirus zoonosis in the tropical rain forests of Africa. Am J Trop Med Hyg 1985;34:781-9.
3. Jezek Z, Fenner F. Human monkeypox. In: JL Melnick, editor. Monographs in virology. Volume 17. Basel: Karger; 1988.
4. von Magnus P, Andersen EK, Petersen KB, Birch-Andersen A. A pox-like disease in cynomolgus monkeys. Acta Pathol Microbiol Scand 1959;46:156-76.
5. Khodakevich L, Jezek Z, Messinger D. Monkeypox virus: ecology and public health significance. Bull World Health Organ 1988;66:747-52.
6. Khodakevich L, Szczeniowski M, Manbu-ma-Disu, Jezek Z, Marennikova S, Nakano J, et al. The role of squirrels in sustaining monkeypox virus transmission. Trop Geogr Med 1987;39:115-22.
7. Fine PE, Jezek Z, Grab B, Dixon H. The transmission potential of monkeypox virus in human populations. Int J Epidemiol 1988;17:643-50.
8. Jezek Z, Grab B, Szczeniowski MV, Paluku KM, Mutombo M. Human monkeypox: secondary attack rates. Bull World Health Organ 1988;66:465-70.
9. Jezek Z, Arita I, Mutombo M, Dunn C, Nakano JH, Szezeniowski M. Four generations of probable person-to-person transmission of human monkeypox. Am J Epidemiol 1986;123:1004-12.
10. Mukinda VB, Mwema G, Kilundu M, Heymann DL, Khan AS, Esposito JJ. Re-emergence of human monkeypox in Zaire in 1996. Lancet 1997;349:1449-50.
11. Centers for Disease Control and Prevention. Human monkeypox-Zaire, 1996-1997. MMWR Morb Mortal Wkly Rep 1997;46:304-7.
12. Mills JN, Childs JE, Ksiazek TG, Peters CJ, Velleca WM. Methods for trapping and sampling small mammals for virologic testing. Atlanta: Centers for Disease Control and Prevention; 1995.
13. Esposito JJ, Massung RF. Poxvirus infections in humans. In: Murray PR, Tenover F, Baron EJ, Pfaller MA, Yolken RH, editors. Manual of clinical microbiology. 6th ed. Washington: ASM Press;1995. p. 1131-8.
14. Towbin H, Staehelin T, Gordon J. Electrophoretic transfer of proteins from polyacrylamide gels to nitrocellulose sheets: procedure and some applications. Proc Natl Acad Sci U S A 1979;76:4350-4.
15. Loparev VN, Parsons JM, Knight JC, Panus JF, Ray CA, Buller RM, et al. A third distinct tumor necrosis factor receptor of orthopoxviruses. Proc Natl Acad Sci U S A 1998;95:3786-91.
16. Ropp SL, Esposito JJ, Loparev VN, Palumbo G. Poxviruses infecting humans. In: Murray PR, Barron CJ, Pfaller MA, Tenover FC, Yolken RH, editors. Manual of clinical microbiology. 7th ed. Washington: ASM Press; 1999. p. 1137-44.
17. Ropp SL, Jin Q, Knight JC, Massung RF, Esposito JJ. PCR strategy for identification and differentiation of smallpox and other orthopoxviruses. J Clin Microbiol 1995;33:2069-76.
18. Esposito JJ, Knight JC. Orthopoxvirus DNA: A comparison of restriction profiles and maps. Virology 1985;143:230-51.
19. Dean AG, Dean JA, Coulombier D, Burton AH, Brendel KA, Smith DC. Epi Info, version 6: A word-processing, database, and statistics program for public health on IBM-compatible microcomputers. Atlanta: Centers for Disease Control and Prevention; 1994.
20. Fenner F, Wittek R, Dumbell KR. The Orthopoxviruses. San Diego: Academic Press; 1989. p. 162-5, 312-15.
21. Ziegler DW, Hutchinson HD, Koplan JP, Nakano JH. Detection by radioimmunoassay of antibodies in human smallpox patients and vaccinees. J Clin Microbiol 1975;1:311-7.
22. Cherry JD, McIntosh K, Connor JD, Benenson AS, Alling DW, Rolfe UT, et al. Primary percutaneous vaccination. J Infect Dis 1977;135:145-54.
23. McIntosh K, Cherry JD, Benenson AS, Connor JD, Alling DW, Rolfe UT, et al. Clinical and serologic study of four smallpox vaccines comparing variations of dose and route of administration. Standard percutaneous revaccination of children who receive primary percutaneous vaccination. J Infect Dis 1977;135:155-66.
24. Jezek Z, Szczeniowski M, Paluku KM, Mutombo M, Grab B. Human monkeypox: confusion with chickenpox. Acta Trop 1988;45:297-307.
25. Centers for Disease Control and Prevention. Human monkeypox — Kasai Oriental, Democratic Republic of Congo, February 1996-
